# Changes in the Biology and Susceptibility of Weevil (*Cylas formicarius*) to the Insecticide Spinetoram as a Response to Cadmium Contamination

**DOI:** 10.3390/toxics12040304

**Published:** 2024-04-22

**Authors:** Jin Xu, An Tang, Jun-Yan Liu, Chang-Long Yao, Ke-Ping Liu, Xiao-Sheng Huang, Pei-Qiong Shi

**Affiliations:** 1 Department of Biotechnology, College of Coastal Agricultural Sciences, Guangdong Ocean University, Zhanjiang 524088, China; xujin@gdou.edu.cn (J.X.); annie.tangan@gmail.com (A.T.); liujunyan@126.com (J.-Y.L.); yaocl3@haid.com.cn (C.-L.Y.); a19875905807@126.com (K.-P.L.); huangxiaosheng@stu.gdou.edu.cn (X.-S.H.); 2 Department of Horticulture, College of Coastal Agricultural Sciences, Guangdong Ocean University, Zhanjiang 524088, China

**Keywords:** cadmium-contaminated sweet potato, *Cylas formicarius*, food intake, survivorship, larval development, spinetoram resistance, *Beauveria bassiana* susceptibility, feeding stimulation

## Abstract

The sweet potato weevil *Cylas formicarius* is a notorious underground pest in sweet potato (*Ipomoea batatas* L.). However, little is known about the effects of cadmium (Cd) stress on weevil biology and resistance to pesticides and biotic agents. Therefore, we fed sweet potato weevils with Cd-contaminated sweet potato and assessed adult food intake and survival and larval developmental duration and mortality rates, as well as resistance to the insecticide spinetoram and susceptibility to the entomopathogenic fungus *Beauveria bassiana*. With increasing Cd concentration, the number of adult weevil feeding holes, adult survival and life span, and larval developmental duration decreased significantly, whereas larval mortality rates increased significantly. However, at the lowest Cd concentration (30 mg/L), adult feeding was stimulated. Resistance of adult sweet potato weevils to spinetoram increased at low Cd concentration, whereas Cd contamination did not affect sensitivity to *B. bassiana*. Thus, Cd contamination affected sweet potato weevil biology and resistance, and further studies will investigate weevil Cd accumulation and detoxification mechanisms.

## 1. Introduction

Heavy metals are defined as metal elements with a density higher than 5 g/cm^3^, generally categorized as either essential or nonessential [1]. Nonessential heavy metals, such as cadmium (Cd), mercury (Hg), lead (Pb), nickel (Ni), and copper (Cu), are highly reactive and toxic at low concentrations, posing severe risks to many organisms, including plants and insects [2,3]. Heavy metal pollution is distributed globally because of human production activities, including mining, smelting, sewage discharge, and use of metal-containing pesticides and fertilizers [4,5,6]. It is estimated that the annual global emissions of Cd, Pb, and Cu reach approximately 1.0 × 10^6^ tons, 5.0 × 10^6^ tons, and 3.4 × 10^6^ tons, respectively [7]. Cadmium can be easily taken up by agricultural crops due to the relatively good solubility of its salts in water, posing a threat to nearly all organisms [3,8,9,10,11,12]. It is estimated that anthropogenic activities release 3–10 times more Cd into the environment than natural processes [1]. In China, the Cd point exceeding rates was 7.0% [13]; the risk screening value for Cd content in non-paddy field soils (PH ≤ 7.5) was 0.3 mg/kg, and it was 0.6 mg/kg for soils with a PH higher than 7.5 [14]. The Cd content in the soils ranged from 0.25 to 0.3 mg/kg in Guangdong province, which is close to the risk screening value [7].

In soil–plant–insect systems, heavy metal accumulations in soil and plants affect both plants and insects. The effects of heavy metals have been examined in a variety of plants (e.g., rice, maize, soybean, kale), as well as in coleopteran, hemipteran, dipteran, lepidopteran, and orthopteran insects [3,6,15,16,17,18,19,20]. Among hypotheses to explain metal hyperaccumulation in plants, the elemental defense hypothesis is the most widely tested, and it proposes that metals accumulate in plant tissues to defend against insect herbivory [3,21,22,23,24]. Esteves-Aguilar et al. [25] reported that heavy metal exposure increases physical and chemical defenses against herbivores in *Wigandia urens* Kunth (Boraginaceae), a shrub or small tree. When fed *W. urens* leaf tissue containing heavy metals, the grasshopper *Sphenarium purpurascens* Charpentier showed higher mortality in the first two developmental instars and lower body biomass than in grasshoppers fed heavy metal-free leaf tissue. According to Jiang et al. [26], Cd stress increased the resistance of larch (*Larix olgensis*) seedlings to gypsy moth, with decreases in gypsy moth body weight and detoxification mechanisms associated with changes in contents or activities of protease inhibitors, tannins, and phenolic acids in *L. olgensis* seedling needles. For insects, heavy metals can be transferred to herbivorous insects through food chains, causing chronic toxicological effects on growth and physiology [27], altering behavior [28], and simplifying the community of plant-associated insects [6,29]. After an initial taste of metal-rich plant materials, the desert locust *Schistocerca gregaria* develops an aversion and decreases ingestion rates [30], whereas the grass miner *Chromatomyia milii* reduces feeding and oviposition on heavy metal-exposed plants [31]. In general, compared with control insects, insects exposed to heavy metals have significantly lower egg viability, larval and pupal survivorship, adult emergence, fecundity, and net reproductive rate [17,31,32]. Recently, the combined effects of heavy metals and other factors on insects have also received attention. Previous studies have shown that heavy metal exposure can induce tolerance (a phenomenon known as “cross-tolerance”) or affect susceptibility of organisms to other stressors [7]. Babczynska et al. [2] reported that polypropylene fibers cause an increase in Cd accumulation in larval *Spodoptera exigua*. Under the combined action of a heavy metal and the pesticide dimethoate, a decrease in grasshopper enzyme activity was particularly notable in areas with low heavy metal concentrations [33]. Heavy metal exposure can also increase insect susceptibility to entomopathogenic microorganisms [27].

Sweet potato (*Ipomoea batatas* (L.) Lam.) is an important food crop worldwide. Its tuberous root is a staple food as well as an important raw material for alcohol and starch manufacturing and food processing, and its stems and leaves are also now a popular vegetable [34]. Sweet potato can adapt to a variety of extreme environmental conditions, such as lack of soil nutrients, drought, and contaminated soil. Some reports showed that sweet potato easily absorbs and accumulates Cd in roots and shoots when grown in contaminated soil [34,35,36,37,38,39]. Although the adaptability of sweet potato to the environment is relatively strong, yield and quality are adversely affected by diseases and insect pests. The sweet potato weevil *Cylas formicarius* Fabricius (Coleoptera: Brentidae) is a notorious pest in sweet potato that causes severe damage to tubers during both growth and storage and thus affects the yield and quality of sweet potato [40,41,42].

Donghai Island in Zhanjiang, Guangdong Province, China, is one of the main areas of sweet potato cultivation; the yield loss caused by sweet potato weevil is generally 5% to 20%, up to 30−50% in severe cases [42]. Chemical control and agronomic measures can protect sweet potato from these weevils, but the occurrence of sweet potato weevil in Donghai Island exhibit an upward trend. In addition, there is a hidden danger of heavy metal pollution on this island due to the steel works in the area. As heavy metals have chronic toxicological effects on insects and can induce cross-tolerance, we hypothesized that the fitness of sweet potato weevil *C. formicarius* may also be affected by heavy metal pollution; therefore, more caution may be necessary in selecting pest prevention and control methods. However, how heavy metal pollution affects sweet potato weevils remains unclear. Therefore, the objectives of this study were (1) to assess the effects of Cd^2+^ on feeding and survival of sweet potato weevils and (2) to evaluate the influence of Cd^2+^ exposure on sweet potato weevil resistance to an insecticide and susceptibility to an entomopathogenic fungus.

## 2. Materials and Methods

### 2.1. Insects, Sweet Potato and Cadmium Concentrations

The *C. formicarius* adults (1-day-old) and 2nd instar larvae used in the study were from laboratory stock reared under standard conditions (26 ± 1 °C and 16:8 h light:dark photoperiod) in the Environmental Protection Laboratory, College of Coastal Agricultural Sciences, Guangdong Ocean University, Zhanjiang, China. The original *C. formicarius* were collected from damaged sweet potatoes in November 2020 from Donghai Island (110°11′−110°21′ E, 20°55′−21°55′ N), Zhanjiang. The tuberous root of sweet potato (*I. batatas* var. *xiguahong*) was used as food for rearing *C. formicarius*, which were commercially available and free of heavy metal contamination. Cadmium chloride (98% CdCl_2_; Macklin, Shanghai, China) solution was used to prepare five Cd treatment concentrations: 30 mg/L, 60 mg/L, 120 mg/L, 240 mg/L, and 480 mg/L, which were determined by pre-experimental screening. Deionized water was the control.

### 2.2. Food Intake by Adult Sweet Potato Weevils

**Figure 1:**
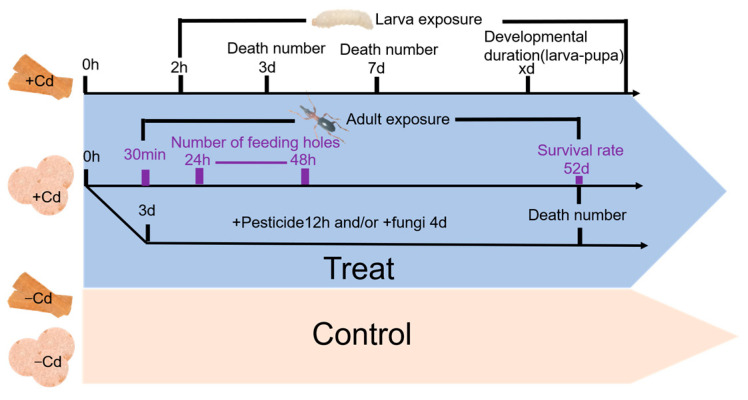
Framework of different experiments in the study.

Adult sweet potato weevils feeding on sweet potato typically form round feeding holes on the surface of sweet potato tuberous root, so weevil food intake can be assessed by the number of feeding holes. Fresh and clean roots of sweet potato were cut into 1 mm-thick slices, and then 3 cm diameter discs were produced with a hole punch. Sweet potato discs were soaked in the different cadmium chloride solutions for 30 min and then air-dried. After 24 h of starvation, vigorous female and male adult sweet potato weevils (1-day-old) were randomly selected and placed in plastic boxes (diameter: 5 cm; height: 3 cm) to feed on the treated potato discs. Each plastic box was a replicate containing 10 male adults and/or female adults and one disc, and there were six replicates per treatment. To prevent drying and mold from developing, potato discs were kept moist with filter paper soaked in the appropriate treatment concentration, with new filter papers and discs treated by the above method used after 24 h. At 24 and 48 h after feeding, the number of holes was recorded. Feeding holes with a 1 mm diameter were denoted by the number 1, whereas those with a diameter less than 1 mm were denoted by the number 0.5. Large feeding holes (areas larger than 1 mm^2^) formed by repeated feeding were denoted by the number 2, 3, or 4 according to the size. The recorded numbers were summed as the number of feeding holes under per treatment. Antifeeding rate (%) was calculated using the following formula: Antifeeding rate (%) = (Number of feeding holes (control) − Number of feeding holes(treatment))/(Number of feeding holes (control)). The adult food intake and other experiments are summarized in Figure 1.

### 2.3. Adult Daily Survival Rate

After 24 h of starvation, vigorous female and male adult (1-day-old) weevils were randomly selected to feed on the treated potato discs. Treatment of potato discs and determination of feeding by adults were the same as those in the section “Food intake by adult sweet potato weevils”, but the numbers of dead females and males were recorded daily until all individuals in a treatment group were dead. There were 60 male and 60 female adults per treatment.

### 2.4. Larval Survival Rate and Developmental Duration

Fresh and clean sweet potatoes were cut into cubes (length: 1.5 cm; width: 1 cm; height: 3 cm), and a hole was excavated in each cube (approximately 0.5 cm × 0.5 cm × 0.5 cm). The sweet potato cubes were soaked in the different cadmium chloride solutions for 2 h and then air-dried. To simulate the larval growth environment, one 2nd instar weevil larva was placed in the hole of each cube, and the hole was sealed with a piece of sweet potato (1 mm thick and treated with the appropriate cadmium chloride solution) fixed with pins. Pupation and death of sweet potato weevil larvae at different concentrations were recorded every 24 h. There were 10 larvae per replicate, with six replicates per treatment.

### 2.5. Resistance of Adult Sweet Potato Weevils to Spinetoram and Susceptibility to Beauveria bassiana

To assay the effects of cadmium chloride on pesticide resistance of adult sweet potato weevils, spinetoram 60 SC (60 g/L SC, US Dow AgroSciences Biological Chemistry Company, Indianapolis, IN, USA) was used in this study. The experiment was conducted in two steps. First, to determine the spinetoram LC_50_ value, spinetoram 60 SC was diluted by 1000, 1500, 2000, 2500, 3000, and 3500 times, the corresponding concentrations were 60 mg/L, 40 mg/L, 30 mg/L, 24 mg/L, 20 mg/L, and 17.14 mg/L, respectively, and deionized water was used as the control. Vigorous adult sweet potato weevils were randomly selected and placed on fresh sweet potato discs (diameter: 3 cm; thickness: 1 mm), which were then treated by dipping in different dilutions of the test formulation for 20 s, followed by and drying on filter paper. Treated adults and sweet potato discs were placed in boxes (diameter: 5 cm; height: 3 cm). The LC_50_ value was calculated from the number of deaths after 12 h. Second, adult weevils were fed on sweet potato discs soaked in the different cadmium chloride solutions for 30 min. After 3 d, vigorous adults were selected and then treated along with sweet potato discs by dipping for 20 s in the LC_50_ dilution concentration of the test formulation. There were 10 adults per replicate, with six replicates per treatment. The number of deaths was counted after 12 h.

*Beauveria bassiana* Bals-1722 strain (GDMCC No: 62530, Guangdong Provincial Microbial Culture Preservation Center, Guangzhou, China) was cultured on potato dextrose agar (PDA) medium under standard conditions (28 °C and 14:10 h light:dark photoperiod). Adult sweet potato weevils were fed on cadmium chloride-treated sweet potato discs for 3 d, and then vigorous adults were treated by dipping in a spore suspension (2.5 × 10^8^ CFU/mL) for 20 s. The control was 0.05% Tween 80. There were 10 adults (gender was randomized) per replicate, with six replicates per treatment. After 4 d, the number of deaths was counted.

### 2.6. Statistical Analyses

Results are reported as the mean ± standard error of the mean. For all ANOVA analysis of adult food intake, larval developmental duration and mortality, and adult resistance to spinetoram and susceptibility to *B. bassiana* assays, data were checked for homogeneity by Levene’s test. Multiple comparisons of means were assessed by Tukey’s HSD test at a significance level of α = 0.05. The difference of food intake between males and females at the same times and/or concentrations was analyzed using an independent-samples t-test, at a significance level of α = 0.05. Adult mortality rates were transformed to probits for linear regression analysis and the determination of spinetoram’ LC_50_ values. The significance of the difference between life span curves (weevils fed on normal food versus weevils fed on metal-supplemented food) was analyzed using a log-rank (Mantel–Cox) statistical test. The log-rank test was performed by GraphPad Prism 9.0.0 (Dotmatics, MA, USA). Probit analysis and other statistical analyses were performed using IBM SPSS Statistics software v.18.0 (IBM, New York, NY, USA).

## 3. Results

### 3.1. Food Intake of Adult Sweet Potato Weevils

**Figure 2:**
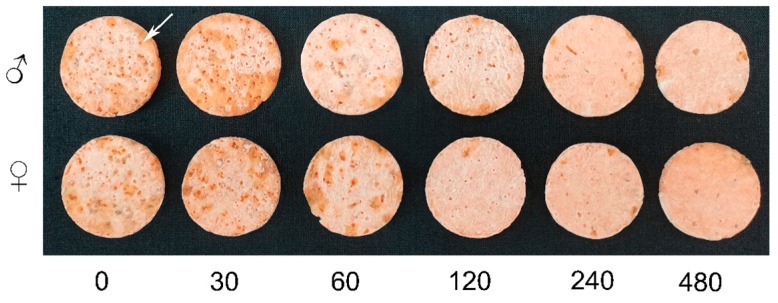
Feeding by adult sweet potato weevils on sweet potato discs treated with different concentrations of cadmium chloride solution (0 to 480 mg/L) at 48 h. The white arrow indicates a feeding hole, sweet potato disc size: 1 mm in thick and 3 cm in diameter.

The number of adult weevil feeding holes on sweet potato discs was significantly affected by Cd concentration (Table 1, Figure 2), with the number decreasing with increasing Cd concentration (*p* ˂ 0.00001). The antifeeding rates of male and female weevils increased significantly with the increase in Cd concentration. Adult weevil food intake decreased significantly at the highest Cd concentrations (240 and 480 mg/L), with numbers of feeding holes less than 10 and antifeeding rates ranging from 87.64% at 24 h to 93.06% at 48 h (*p* ˂ 0.00001). Notably, at 48 h, compared with the control, the number of feeding holes increased at the Cd concentration of 30 mg/L, although not significantly. However, the numbers of male and female feeding holes at 30 mg/L were significantly higher than those at 60, 120, 240, and 480 mg/L (*p* ˂ 0.00001). Similarly, compared with the control, male and female antifeeding rates at 30 mg /L were −15.93% ± 6.81% and −12.35% ± 8.87%, respectively. There were no significant differences in food intake between males and females at the same times and concentrations (*p* ˃ 0.05).

### 3.2. Adult and Larval Survival Rates and Larval Developmental Duration

**Figure 3:**
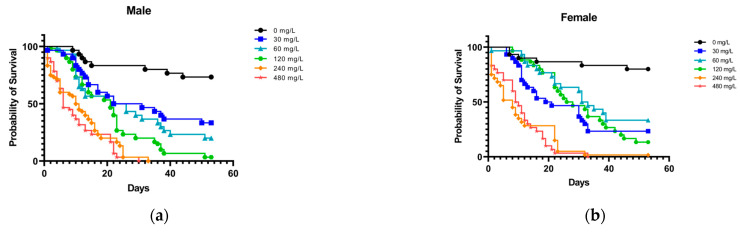
Survival rates of (**a**) male and (**b**) female sweet potato weevils fed on sweet potato discs treated with different Cd concentrations (cadmium chloride: 0 to 480 mg/L).

Survival rates of male and female weevils fed on sweet potato discs soaked in different concentrations of cadmium chloride solution after 52 d are shown in Figure 3a,b. The survival curves of males and females were significantly different among Cd treatments (males: χ^2^ = 194, df = 5, *p* ˂ 0.0001; females: χ^2^ = 233.5, df = 5, *p* ˂ 0.0001). Male survival rates at 52 d were 73.33% ± 3.85% at 0 mg/L, 33.33% ± 5.09% at 30 mg/L, 20% ± 11.55% at 60 mg/L, and 3.33% ± 1.92% at 120 mg/L (Table 2). Female survival rates at 52 d were 80% ± 8.82% at 0 mg/L, 23.33% ± 10.72% at 30 mg/L, 30% ± 11.55% at 60 mg/L, and 13.33% ± 5.09% at 120 mg/L (Table 2). At 240 and 480 mg/L, approximately half of females and males died within 5 to 10 d, and all treated individuals died within 30 to 34 d. Notably, the survival of female adults at 30 mg/L was lower than that at 60 and 120 mg/L (Figure 3b).

**Figure 4:**
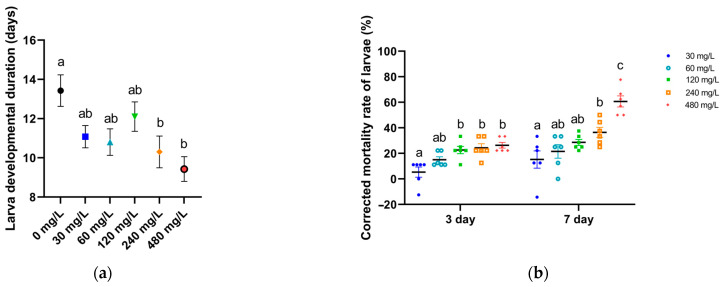
Sweet potato weevil larval (**a**) developmental duration (mean ± SE) and (**b**) corrected mortality (mean ± SE, *n* = 6) after feeding on sweet potato discs soaked in different concentrations of cadmium chloride solution (0 to 480 mg/L). Feeding times: 3 and 7 d. Different lowercase letters denote significant differences among treatments according to Tukey’s HSD.

The effects of cadmium chloride on weevil larval developmental duration and survival were also determined. Developmental duration decreased significantly with the increase in Cd concentration (*p* = 0.002, F_5,234_ = 3.8975), with duration 13.43 ± 0.80 d at 0 mg/L, 11.08 ± 0.57 d at 30 mg/L, 10.8 ± 0.68 d at 60 mg/L, 12.1 ± 0.75 d at 120 mg/L, 10.3 ± 0.81 d at 240 mg/L, and 9.43 ± 0.63 d at 480 mg/L (Figure 4a, Table 3). The corrected mortality rates of weevil larvae increased significantly with the increase in Cd concentration and feeding time (Figure 4b, Table 3). At day 3, the corrected mortality rate was 5.32% ± 4% at 30 mg/L, which increased significantly to 22.68% ± 2.91% at 120 mg/L, 24.31% ± 3.24% at 240 mg/L, and 26.39% ± 2.24% at 480 mg/L (*p* = 0.0002, F_4,25_ = 8.2651). At day 7, the corrected mortality was 15.21% ± 6.73% at 30 mg/L, which increased significantly to 36.47% ± 3.88% at 240 mg/L and 60.68% ± 4.37% at 480 mg/L (*p* ˂ 0.000001, F_4,25_ = 13.7343).

### 3.3. Resistance of Adult Sweet Potato Weevils to Spinetoram and Susceptibility to Beauveria bassiana

**Figure 5:**
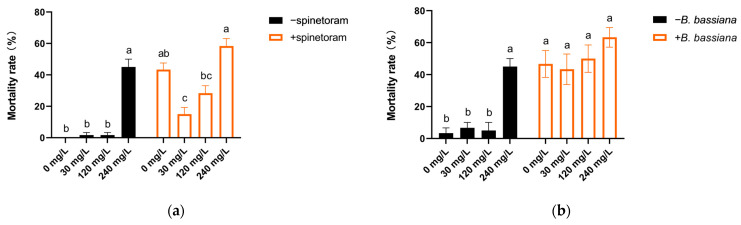
Adult sweet potato weevil (**a**) resistance to the insecticide spinetoram and (**b**) sensitivity (mean ± SE, *n* = 6) to the entomopathogenic fungus *Beauveria bassiana* after feeding on Cd-contaminated sweet potato discs. Different lowercase letters denote significant differences according to Tukey’s HSD.

The susceptibility of adult sweet potato weevils to spinetoram 60 SC is shown in Table 4 and Figure 5a. The corrected mortality of adult sweet potato weevils increased significantly with the increase in concentration of spinetoram (Table 4; *p* ˂ 0.0001, F_5,30_ = 37.794). The concentration (μg/mL) that caused 50% mortality (LC_50_) of adults was 22 mg/L (χ^2^ = 7.069, *p* = 0.132). At the spinetoram concentration of LC_50_, resistance of sweet potato weevils to spinetoram varied with the Cd treatment concentration of the sweet potato discs. The mortality rate at 30 mg/L was significantly lower than that at 0 and 240 mg/L (Figure 5a; *p* ˂ 0.0001, F_3,20_ = 17.1769). After feeding on Cd-contaminated sweet potato discs, the sensitivity of sweet potato weevils to *B. bassiana* was not affected (Figure 5b; *p* = 0.3628, F_3,20_ = 1.1247).

## 4. Discussion

Heavy metal exposure can modify growth and development and alter sensitivity to other biological and abiotic factors in many species [27,43]. Many invertebrates, including insects, are therefore good bio-indicators for assessing the effects of heavy metals [18,44]. In this study, bioassays were used to assess the effects of Cd stress on sweet potato weevil feeding amounts, growth and survival, and susceptibility to the pesticide spinetoram and the entomopathogenic fungus *B. bassiana*.

When living in contaminated environments or after an initial taste of metal-rich plant materials, insects develop an aversion and decrease ingestion rates [17,28]. In this study, as Cd concentration increased, food intake of adult sweet potato weevils decreased, which is consistent with previous reports. For example, the desert locust *S. gregaria* prefers leaves and artificial food with low Zn concentrations [30]. The thrip *Frankliniella occidentalis* decreases feeding when reared on plants exposed to Cd, compared with those fed noncontaminated plants [45]. However, in this study, the cadmium contents in sweet potato chips and weevils were not determined for all treatments, which need to be further clarified in the subsequent studies on Cd accumulation. In addition to appetite, heavy metals have other toxic effects on insects; in severe cases, they have adverse effects on the growth index, development duration, and mortality rate [15,46,47]. Exposure of insects to Cd^2+^ can delay development [28,32]. Luo et al. [48] reported that Cd in a diet inhibited development of the Asian corn borer *Ostrinia furnacalis* by extending the duration of larval and pupal stages and decreased the survival rate. In this study, larval mortality rates increased significantly with the increase in Cd concentration and feeding time, whereas the duration of larval development (2nd instar larva to pupa) decreased with increasing Cd concentration. Because the pupal stage can help insects withstand adversity, the accelerated pupation of sweet potato weevils might be an adaptive strategy to overcome Cd toxicity. Whether this strategy requires additional biological costs, such as decreases in pupal weights, eclosion rates, and adult fecundity, needs further study. This may also be related to insect species, as the ability to detox heavy metals varies among different insects [4,49,50]. In this study, for adult weevils, survival rate and life span generally decreased with increasing Cd concentration, which were consistent with the effects of Cd on other insects (e.g., *Drosophila melanogaster*, moth of cotton bollworm *Helicoverpa armigera*, *Spodoptera litura*) [17,51,52]. Adults ate less from sweet potato highly contaminated with Cd^2+^, which can also reduce survival rates and shorten life spans. The phenomenon of heavy metal toxicity stems from the entirety of insect’s biochemical processes, which usually involve the special immune and enzyme detoxification systems of insects [15,53,54]. Wu et al. [54] reported that Cd exposure decreases both cellular immunity and humoral immunity of gypsy moth larvae (*Lymantria dispar*); the total hemocyte count and immune activity of hemolymph in gypsy moth larvae exposed to Cd stress were significantly decreased, and signal transduction genes and antimicrobial peptide genes in the Toll, IMD, and JAK/STAT signaling pathways were also significantly decreased in Cd-treated larvae. Insect detoxifying enzymes (e.g., glutathione S-transferases (GSTs), carboxylesterase (CarE), acetylcholinesterase (AChE)) play important roles in detoxifying toxic heavy metals [53]. In adult *Drosophila*, GstD2 and GstD6 were induced upon Cd exposure, thereby strengthening the defense ability of adult *Drosophila* against toxic metal exposure [55]. However, dietary Cd inhibited the activities of GST, CarE and P450 in *Helicoverpa armigera* [51]. In addition, metallothioneins (Mtns) in insects were increased upon exposure to Cd, Pb, and Hg, respectively [55,56]. The detailed mechanisms involved in the responses of sweet potato weevil to Cd exposure are worthy of further study.

Notably, sweet potato treated with the lowest Cd concentration of 30 mg/L stimulated feeding of adult sweet potato weevils after 48 h. However, the probability of female survival was lower in the 30 mg/L treatment than in 60 and 120 mg/L treatments. To explain this result, we hypothesized that females might eat more food at low Cd concentrations because they needed energy to lay eggs or that stimulation of feeding at the low concentration lasted longer in females than in males. Whether the stimulation of feeding is a compensatory response after starvation or an adaptation to low Cd concentration and how long the effect lasts in males and females need further study. Van Ooik and Rantala [16] reported that heavy metal pollution increased immune function in female autumnal moths (*Epirrita autumnata*) but not in males, and Butovsky [18] reported that accumulation of heavy metals, including Cd, Zn, and Cu, was sex-specific in carabid beetles. Thus, we hypothesized that the survival rate was also related to immune function and Cd accumulation in female and male sweet potato weevils under Cd stress.

Heavy metal exposure can affect susceptibility of organisms to other stressors [57,58]. For example, heavy metal exposure may increase insect susceptibility to entomopathogenic microorganisms [27]. Resistance of larvae of the greater wax moth *Galleria mellonella* to the fungus *B. bassiana* decreased when larvae were exposed to Ni at a sublethal level [59]. Similarly, the susceptibility of *Drosophila melanogaster* to *Bacillus subtilis* increased in a dose–response fashion with exposure to Pb [60]. In this study, the sensitivity of adult sweet potato weevils to *B. bassiana* was not affected by Cd contamination, which might be due to differences in insect species and fungal strains. Heavy metal exposure can also affect insect resistance to pesticides. After exposure to dimethoate, grasshoppers (*Chorthippus brunneus*) collected from industrial areas polluted with heavy metals had different metabolic reactions compared with those collected from unpolluted sites, with activity of detoxification enzymes particularly affected [33]. In the beet armyworm *S. exigua*, tolerance to the pesticide spinosad increased after long-term selection by Cd [43]. In this study, the resistance of adult sweet potato weevils to spinetoram increased after exposure to a low level of Cd^2+^, which could contribute to reduced efficiency of pesticide-based plant protection efforts. Tolerance development may involve behavioral resistance, penetration resistance, target resistance, and metabolic resistance [61], but the exact mechanism of cross-tolerance formation is not fully understood; the molecular mechanism of this enhanced tolerance also deserves further study.

Evaluation of the impacts of heavy metal exposure on herbivorous insects is particularly important for assessing eco-toxicological risk in food chains [46]. In addition, the development of insect heavy metal tolerance has probably led to the increase in their tolerance to pesticides [7,43], which may reduce the efficiency of pest chemical control and bring new challenges to pest control in the farmland with heavy metal pollution [46]. In this study, weevil resistance to spinetoram increased after exposure to a low level of Cd^2+^, but the sensitivity to *B. bassiana* was not affected by Cd contamination. Microbial control may be more suitable for pest control in heavy metal-polluted areas than chemical control [7]. Admittedly, the cross-tolerance was tested under controlled conditions, which enhances our understanding of the effects of single heavy metal on insects but may not accurately reflect the characteristics of natural communities because heavy metal pollution in the field generally involves a variety of heavy metals, and their impacts on insects are more diversified and complicated [46,56,57]. Therefore, it is more meaningful to combine indoor experiments with field investigations to explore the insect resistance or tolerance under heavy metal exposure.

In summary, when sweet potato weevils were fed Cd-contaminated diets, larval developmental duration and adult feed intake, survival, and life span decreased with increasing Cd concentration. However, weevil resistance to spinetoram increased after exposure to a low level of Cd^2+^. The findings provide a basis to further explore the effects of heavy metals on weevils. To better understand the effects of heavy metals, we plan to conduct further studies on sweet potato weevil Cd accumulation and detoxification mechanisms using indoor experiments and field investigations.

## Figures and Tables

**Table 1:** Numbers of feeding holes and antifeeding rates of adult sweet potato weevils on sweet potato discs treated with different concentrations of CdCl_2_.

Concentration of CdCl_2_ (mg/L)	24 h				48 h			
	Male		Female		Male		Female	
	Number of Feeding Holes	Antifeeding Rate (%)	Number of Feeding Holes	Antifeeding Rate (%)	Number of Feeding Holes	Antifeeding Rate (%)	Number of Feeding Holes	Antifeeding Rate (%)
0	62.67 ± 5.68 a	− −	76.83 ± 2.54 a	− −	71.67 ± 4.67 a	− −	86 ± 5.34 a	− −
30	30.08 ± 2.58 b	52.00 ± 4.11 b	32.17 ± 2.26 b	58.13 ± 2.94 c	83.08 ± 4.88 a	−15.93 ± 6.81 d	96.62 ± 7.63 a	−12.35 ± 8.87 d
60	12.92 ± 2.08 c	79.39 ± 3.31 a	18.75 ± 2.94 c	75.60 ± 3.82 b	43.83 ± 5.35 b	38.84 ± 7.47 c	56.00 ± 5.09 b	34.88 ± 5.92 c
120	9.67 ± 1.25 c	84.58 ± 2.00 a	10.5 ± 1.39 cd	86.33 ± 1.81 a	21.25 ± 2.76 c	70.35 ± 3.85 b	24.25 ± 2.24 c	71.80 ± 2.60 b
240	7.00 ± 0.37 c	88.83 ± 0.58 a	9.33 ± 0.77 d	87.85 ± 1.00 a	7.67 ± 0.28 bcd	89.30 ± 0.39 ab	7.17 ± 0.33 c	91.18 ± 0.48 ab
480	7.50 ± 0.37 c	88.03±0.58 a	9.5 ± 0.37 d	87.64 ± 0.48 a	5.33 ± 0.25 d	93.06 ± 0.32 a	6.00 ± 1.13 c	93.02 ± 1.32 a
*p*	˂0.00001	˂0.00001	˂0.00001	˂0.00001	˂0.00001	˂0.00001	˂0.00001	˂0.00001
df	5.30	4.25	5.30	4.25	5.30	4.25	5.30	4.25
F	63.54	35.99	182.12	29.44	79.98	86.96	79.12	81.43

Note: Different lowercase letters within the same column indicate significant differences among treatments based on one-way ANOVA with Tukey’s HSD test at significance level α = 0.05. Feeding times: 24 and 48 h. Values are the mean ± standard error (*n* = 6). Antifeeding rate (%) was calculated as the ratio of the difference between the feeding holes of control and treatment to the feeding holes of control.

**Table 2:** Survival rates of male and female weevils fed on sweet potato discs treated with different Cd concentrations (after feeding for 52 days).

Concentration of CdCl_2_ (mg/L)	Male Survival Rates (%)	Female Survival Rates (%)
0	73.33 ± 3.85 a	80 ± 8.82 a
30	33.33 ± 5.09 b	23.33 ± 10.72 b
60	20 ± 11.55 bc	30 ± 11.55 b
120	3.33 ± 1.92 c	13.33 ± 5.09 b
240	0c	0 b
480	0 c	0 b

Note: Different lowercase letters indicate significant differences among treatments based on one-way ANOVA with Tukey’s HSD test at significance level α = 0.05.

**Table 3:** Sweet potato weevil larval developmental duration and corrected mortality after feeding on sweet potato discs soaked in different concentrations of cadmium chloride solution.

Concentration of CdCl_2_ (mg/L)	Larva Developmental Duration (d)	Larva Corrected Mortality Rates (%)	
		Day 3	Day 7
0	13.43 ± 0.8 a	——	——
30	11.08 ± 0.57 ab	5.32 ± 4 a	15.21 ± 6.73 a
60	10.8 ± 0.68 ab	15.05 ± 2.28 ab	21.53 ± 5.32 ab
120	12.1 ± 0.75 ab	22.68 ± 2.91 b	28.60 ± 2.37 ab
240	10.3 ± 0.81 b	24.31 ± 3.24 b	36.47 ± 3.88 b
480	9.43 ± 0.63 b	26.39 ± 2.24 b	60.68 ± 4.37 c

Note: Different lowercase letters indicate significant differences among treatments based on one-way ANOVA with Tukey’s HSD test at significance level α = 0.05.

**Table 4:** Resistance of adult sweet potato weevils to spinetoram 60 SC.

Concentration of Spinetoram (mg/L)	Corrected Mortality Rate (%)	Probit	χ^2^	*p*-Value	LC_50_ (mg/L)	95% Confidence Limits (mg/L)
17.14	23.3333 ± 4.9441 e	−5.4081 + 4.0187 x	7.069	0.132	22.16842	17.6107–25.9261
20	45.0000 ± 4.2817 d					
24	60.0000 ± 3.6515 cd					
30	73.3333 ± 4.2164 bc					
40	86.6667 ± 4.9441 ab					
60	91.6667 ± 3.0732 a					

Note: Different lowercase letters indicate significant differences among treatments based on one-way ANOVA with Tukey’s HSD test at significance level α = 0.05. Adult mortality rates were transformed to probits for linear regression analysis and the determination of spinetoram’ LC_50_ values.

## Data Availability

Data is contained within the article.
